# Canine Demodicosis in Rupandehi Nepal’s Street Dogs: Prevalence, Clinical Signs, and Hematology

**DOI:** 10.3390/vetsci12030238

**Published:** 2025-03-03

**Authors:** Rachana Bhusal, Tulsi Ram Gompo, Tatsuki Sugi, Masahito Asada, Kishor Pandey

**Affiliations:** 1 Central Department of Zoology, Institute of Science and Technology, Tribhuvan University, Kathmandu 44601, Nepal; rachana.bhusal123@gmail.com; 2 Central Veterinary Laboratory, Kathmandu 44600, Nepal; tulsigompo@gmail.com; 3 Division of Collaboration and Education, International Institute for Zoonosis Control, Hokkaido University, Sapporo 001-0020, Japan; 4 National Research Center for Protozoan Diseases, Obihiro University of Agriculture and Veterinary Medicine, Obhiro 080-8555, Japan; masada@obihiro.ac.jp

**Keywords:** canine demodicosis, *Demodex* mites, prevalences, hematological changes, rupandehi, Nepal

## Abstract

Canine demodicosis is a contagious skin disease caused by the over-proliferation of *Demodex* mites in the host’s hair follicles. This study examines the prevalence, clinical signs, and hematological changes associated with demodicosis in street dogs of Rupandehi, Nepal. Between August 2023 and January 2024, 100 skin scrapings were collected from each street dog presenting dermatological symptoms. The samples, treated with 10% KOH and microscopically examined, revealed a 21% positivity rate for demodicosis, with all cases involving *Demodex canis*. The infection predominantly affected young puppies (37.5%), females (21.6%), mixed breeds (33.3%), and dogs with above-ideal body conditions (25%). There was no significant association between infection and variables such as age, gender, breed, or nutritional status. Clinically, all affected dogs exhibited alopecia, primarily on the legs. Hematological assessments indicated significant increases in neutrophils and eosinophils and a notable decrease in mean corpuscular hemoglobin concentration and lymphocytes among infected dogs (*p* < 0.05). This study underscores the importance of vigilant monitoring and comprehensive diagnostic practices in effectively managing and treating canine demodicosis, especially in street dogs.

## Simple Summary

Canine demodicosis is a skin disease in dogs that is caused by microscopic mites infesting hair follicles. This study examined the prevalence, clinical symptoms, and blood parameters of this disease among street dogs in Rupandehi, Nepal, and how this mite affected their health. Skin samples were taken from 100 street dogs with skin problems between August 2023 and January 2025. Microscopic examination of those samples revealed that 21% of the dogs were infested with *Demodex canis* mites. The condition was more likely to be seen in puppies, female dogs, cross-breed dogs, and dogs with higher than ideal body weight. Affected dogs had an evident loss of hair on their legs. Blood tests indicated increased levels of neutrophils and eosinophils, signaling an immune response, and a decrease in mean corpuscular hemoglobin concentration. These findings highlight the significance of intensive monitoring and diagnosis of demodicosis among street dogs to improve their health and well-being.

## 1. Introduction

Dogs are prone to skin problems, with ectoparasites such as mites, fleas, ticks, and lice accounting for a major share of these issues and having a significant role in both itchy and non-itchy skin conditions in them [1]. Among these, *Demodex* mites are of particular importance because they cause canine demodicosis, an illness characterized by mild cutaneous inflammation to severe secondary infection, leading to systemic illness or even death [2]. The prevalence of canine demodicosis varies globally, and its occurrence among street dogs, particularly in Nepal, remains underexplored.

Canine demodicosis occurs when an excessive population of *Demodex* mites proliferates within a dog’s hair follicles, usually due to a weak immune response [3,4]. Leydig first described *Demodex canis* in 1859 and further redescribed and reevaluated by Nutin and Desch in 1978 [5,6]. The life cycle of *Demodex* mites, which spans approximately 14–21 days, involves egg deposition by females in the infested hair follicles, followed by larval and nymphal stages before reaching adulthood [7,8]. A variety of internal and external variables affect dogs’ susceptibility to demodicosis. Intrinsic factors include inherited susceptibility, changes in skin structure and biochemistry, immunological illnesses, breed, and age. Extrinsic influences include nutrition, stress levels, and the presence of other underlying disorders or pathogens [9]. *Demodex* mites are classified into three species: *Demodex canis*, *D. injai*, and *D. cornei* each of which lives in various parts of the dog’s body. As a result, infected dogs exhibit a variety of symptoms including skin redness, rash, hair loss, itchiness, licking, scratching, biting, chewing at affected areas, pustules, abnormal pigmentation, skin thickening, and skin feeling like elephant skin [10,11]. Localized or generalized erythema, alopecia, and scales are common clinical symptoms of papular dermatitis. Primary lesions include pustules and papules, whereas erythema and alopecia are the most common secondary lesions, which are typically found on the face, neck, and limbs, with occasional lesions appearing on the trunk of affected dogs [12,13].

Street dogs in Nepal are a neglected population suffering from various skin diseases, including demodicosis [14]. Since veterinary care for these dogs is limited, understanding the prevalence and impact of this disease is essential to improve animal welfare and develop effective treatment strategies. This study is designed to determine the prevalence, clinical presentation, and hematological alterations in street dogs infected with demodicosis in Rupandehi, Nepal. By providing region-specific data, this study is designed to help improve diagnostic efficiency and guide effective treatment strategies for local veterinarians.

Canine demodicosis occurs in two basic forms: localized and generalized, which are further classified according to the age of onset. Localized demodicosis is often characterized by one or two hairless patches on the nose, face, legs, and eyes, most common in young pups under one year of age, and in most cases experience spontaneous remission without the need for medical treatments. Generalized demodicosis, on the other hand, consists of five or more lesions spread over the body and is distinguished by scattered patches, erythematous lesions, scales, and papules [15]. Secondary superficial and deep pyoderma can cause crusting and ulcers on the affected skin. Generalized demodicosis can affect both young and adult dogs, with lesions extending throughout the body. The prognosis of generalized demodicosis is poorer, especially in immunocompromised dogs [16].

Hematological changes are frequently observed in dogs with demodicosis reflecting critical insights into disease severity and immune response. Infected dogs commonly exhibit a considerable drop in the value of hemoglobin and leukocytosis because of the elevation of neutrophils, eosinophils, and lymphopenia [17]. Additionally, significant reductions in erythrocyte counts and increased total leukocyte counts have been reported in affected dogs [18]. These alterations are potential for diagnosis use in determining the severity of disease and can aid in treatment planning.

Despite the prevalence of skin diseases being high among street dogs, there have not been any studies assessing the prevalence of demodicosis in Rupandehi, Nepal. This study makes an effort to fill the gap by estimating its prevalence, clinical presentation, and associated hematological alterations. Understanding the epidemiology of canine demodicosis in street dogs will provide baseline data for future research and veterinary interventions. Additionally, incorporating hematological analysis alongside clinical evaluations may enhance diagnostic accuracy and effective treatment protocols.

## 2. Materials and Methods

### 2.1. Ethical Consideration and Safety Issues

Ethical approval for this research was obtained from the Nepal Veterinary Council, the official veterinary regulating body, with Ref. no. 39/2080/81. Veterinary technicians were fully informed about their roles before the field visits and sample collection time. All the dogs were handled humanely during the time of the study. The dogs were restrained by veterinary technicians only at the time of sample collection for skin scraping and blood collection.

### 2.2. Study Area

**Figure 1:**
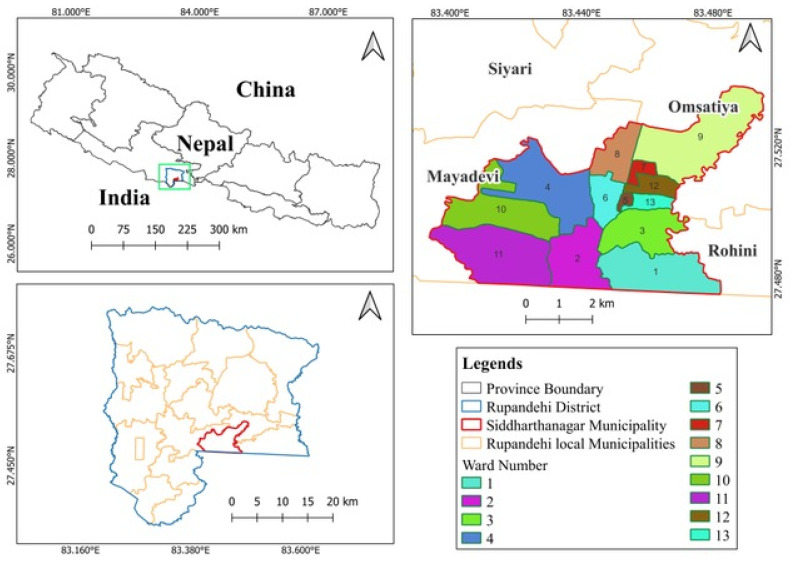
Map illustrating the geographic location of the study area.

This study was conducted in the Siddharthanagar municipality, the administrative headquarters of the Rupandehi district in Lumbini province, Nepal. The Siddharthanagar municipality covers 36.03 square kilometers and is divided into 13 wards (Figure 1).

### 2.3. Sample Selection

This study was conducted from August 2023 to January 2024. We purposefully enrolled 100 street dogs roaming in the street during the study time because there were no precise estimates of the number of stray dog populations from study areas. Dog availability and sample sizes were not evenly distributed each month. The selected dogs have various dermatological problems like alopecia, erythema, hot spots, pigmentation, pustular, and white scales. Age, gender, breed, nutritional status, dermatitis form, type (localized or generalized), and dermatitis area were all simultaneously recorded.

The dog’s age was determined by assessing their growth phases and dental condition [19], whereas the nutritional status of dogs was determined by body condition score, which is a technique for evaluating a dog’s total body composition and nutritional status. Dogs were split into three categories based on their body weight: under-ideal, ideal, and over-ideal [20]. Widespread skin lesions that cover a large area of the body are classified as generalized forms, whereas one or two skin lesions that are limited to certain body parts are classified as localized forms [15].

### 2.4. Dog Identification Approach

To limit the possibility of sample repetition and to ensure the accuracy of data, a simple dog identification technique was used. Photographs of each dog’s front, side, and back were captured, focusing on distinctive physical features, especially coat color patterns. Unique coat patterns (coat color, markings, and any distinctive spots or patches) were carefully observed and recorded. Gender was also determined and noted as an identifier.

### 2.5. Control (Healthy) Dog Group

An extra 10 dogs with ideal nutritional status and no dermatological signs were selected as a control group. Blood samples from these control dogs were collected specifically to facilitate a hematological comparison with the diseased dogs. Including this control group allowed for a clear differentiation of hematological parameters between healthy and diseased dogs, providing valuable insights into the changes associated with canine demodicosis. The physical examination and health screenings were conducted by experienced veterinarians before enrolling in the study which would minimize related confounding factors. These screenings were designed to rule out known parasites and microorganisms that could influence the results.

### 2.6. Intervention Applied

All the sampled dogs exhibited some dermatological signs and almost every sample was infested with either ticks or fleas, indicating a need for immediate treatment. To address this, each sampled dog within the study received a single subcutaneous injection of ivermectin (0.02 mg per kg). This strategy was adopted to ensure that all dogs receive at least one effective dose of medication to provide immediate relief and reduce the burden of external parasites. Given the logistical challenges of administering a complete treatment course to each sampled street dog, a single-dose approach was chosen to maximize impact while under fieldwork conditions.

### 2.7. Sample Collection Technique

#### 2.7.1. Skin Scraping

With the assistance of a veterinary professional, the dog was initially restrained manually. Hair from the sample collection site was cut or clipped using a scissor and the skin was squeezed with the thumb and index fingers of the left hand to help expel the mites from the deep follicles to the surface. Multiple skin scrapings (about 1 cm) of different locations in the direction of hair growth were made with a scalpel blade dipped in mineral oil until bleeding occurred, as mineral oil aids in easy scraping and improved attachment of the collected material (mites) to the scraping blade. Skin scraping, together with the scraping blade, was then stored in a vial with a unique code number and transported to the laboratory for further processing.

#### 2.7.2. Blood Collection

At first, the dog was restrained gently and cooperatively. A small area of fur was shaved from the dog’s leg. This shaved area was also taken as a temporary, visible marker indicating that a skin and blood sample had already been taken from that individual. A blood sample of 2 mL was drawn from the cephalic vein and collected in an EDTA tube. The sample was then transported to Institute of Agriculture and Animal Science (IAAS), Paklihawa Campus, Rupandehi, maintaining a cold chain for further laboratory investigations of hematological parameters such as hemoglobin (Hb), red blood cells (RBC), platelets, packed cell volume (PCV), mean corpuscular volume (MCV), mean corpuscular hemoglobin (MCH), mean corpuscular hemoglobin concentration (MCHC), white blood cells (WBC), neutrophils, lymphocytes, monocytes, eosinophils, and basophils.

### 2.8. Sample Processing

#### 2.8.1. Skin Scraping and Microscopic Examination

The scraping was placed in the test tube and digested with 10% KOH until totally above the sample. Then, the solution was gently heated (near boiling) with frequent shaking for about 5–10 min until all the debris was digested. After that, the solution was allowed to cool and centrifuged at 1000× *g* for 10 min. A few drops of sediment from the skin scraping were placed on the slide and covered with the cover slip, and the slide was observed under a low and high power (100×, 400×) microscope. This determined the presence or absence of the *Demodex* mites within the sample collected.

#### 2.8.2. Hematological Analysis

An automated hematology analyzer (Mindray Animal Care, Shenzhen, China) was used to analyze the hematological parameters in all the collected blood samples.

#### 2.8.3. Species Identification

Species of *Demodex* were identified by micrometry analysis along with descriptive morphological features of lengths of opisthosoma, podosoma, and gnathosoma and were assessed by using ImageJ software version 1.53. Furthermore, the mean (length and width) of each part and the mean total length of mites (*n* = 21) were listed and compared with standard reference. The ratio of opisthosoma length to body length (%) was determined using the following formula. Ratio (%) = length opisthosoma × 100/total body length. Species were identified under a microscope using published papers [6,21,22].

### 2.9. Statistical Analysis

The raw data were first recorded in a Microsoft Excel spreadsheet. The data contains the animal characteristics as predictors, such as age, gender, breed, and nutritional status, as well as the test outcome indicating either positive or negative demodicosis. The prevalence estimates of the positive cases and the associations of predictor variables with the outcome were assessed using the “epitools” package in R Studio (version 2024.04.2). Any variables with Fisher’s exact *p*-value ≤ 0.05 were considered statistically significant during the analysis. A *t*-test was conducted to analyze and compare the mean hematological parameters between the control group and the group of dogs with demodicosis.

## 3. Results

**Figure 2:**
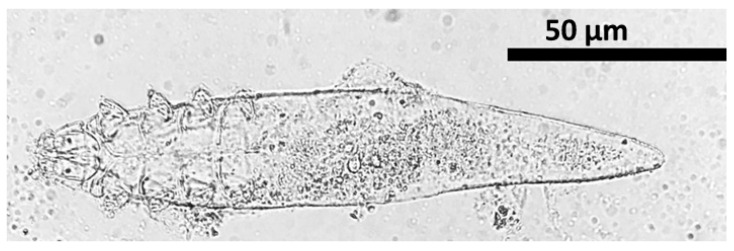
Identified *Demodex* mite species, *Demodex canis*.

Out of 100 dogs with dermatological disorders, 21 (21%, 95% CI: 14.17–29.98) of them tested positive for canine demodicosis. The morphometry analysis of *Demodex* species was presented in Supplementary Table S1. Gnathosoma, podosoma, opisthosoma, and total body length were 18.11 ± 1.4 μm (15.2 to 20.1), 63.03 ± 1.07 μm (60.7 to 64.7), 130.89 ± 4.18 μm (119.8 to 140.1), and 212.04 ± 5.12 μm (197 to 219.8) in that order (Supplementary Table S1). According to the results, the species was *Demodex canis* (Figure 2).

The highest prevalence of demodicosis was reported in young dogs 3/8 (37.50%, 95% Confidence Interval (CI): 13.68–69.42), followed by (>6 years) senior dogs 2/10 (20%, 95% CI: 5.66–50.98). The adult and senior dogs had higher odds of detecting demodicosis compared with the young dogs, but the result was not statistically significant (*p* > 0.05) (Table 1).

Out of 51 female dogs investigated, 11 (21.57%, 95% CI: 12.49–34.63) tested positive for *Demodex* mites. Similarly, 10/49 (20.41%, 95% CI: 11.48–33.64) of the 49 male dogs evaluated tested positive. However, the OR of 0.95 (95% CI: 0.42, 2.75) suggests that female dogs had 0.95 times lower odds of testing positive compared with male dogs, but this result was not statistically significant (*p* = 1).

Out of 91 local breed dogs, 18 (19.78%, 95% CI: 12.89–29.11) were tested positive for *Demodex* mites. Similarly, 3/9 mixed breeds tested positive (33.33%, 95% CI: 12.89–29.11). The OR for mixed breed dogs testing positive compared with local breed dogs was 1.65 (95% CI: 0.53, 8.64) showing no statistically significant difference (*p* = 0.39).

Twenty-one *Demodex*-positive dogs were grouped into under-ideal, ideal, and over-ideal categories based on their body condition scores, indicating their nutritional status. Out of 41 dogs “under-ideal” dogs in body category, 9 were positive for demodicosis (21.95%, 95% CI: 12.00–36.71); out of 43 ideal body categories, 8 were positive (18.60%, 95% CI: 9.74–32.62); and 4 out of 16 “over-ideal” body condition dogs were positive (25.00%, 95% CI: 10.2–49.49). The differences in positive cases percentages between the under-ideal group and the other group were not statistically significant (ideal group vs. under-ideal: OR: 1.23, *p* = 0.79; over-ideal vs. under-ideal: OR: 0.83, *p* = 1).

**Figure 3:**
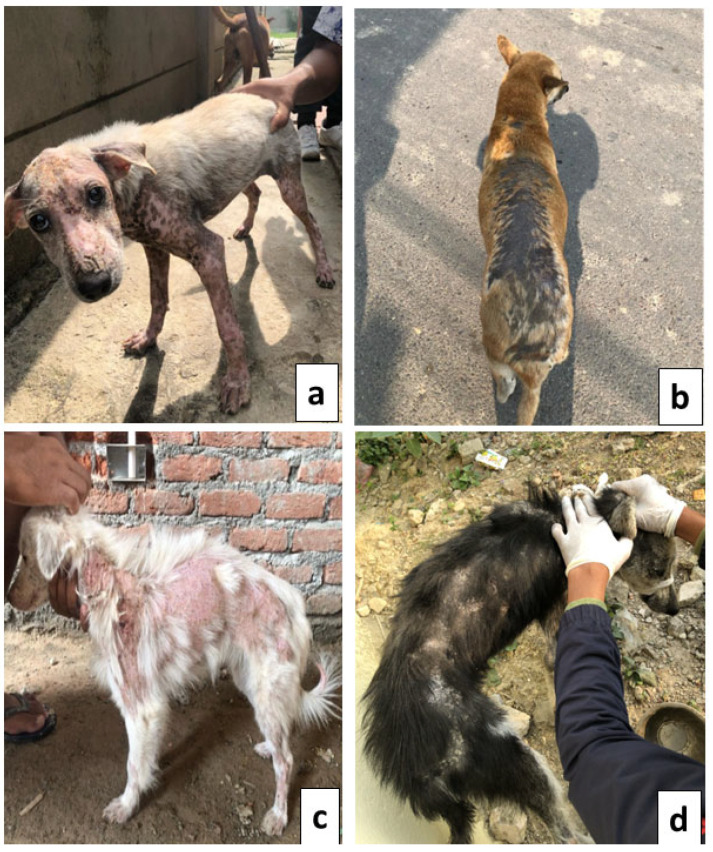
*Demodex*-positive cases in street dogs show different dermatological signs: alopecia and pustule (**a**), alopecia along with hyper-pigmentation (**b**), alopecia with erythema (**c**), alopecia with heavy white scale (**d**).

Out of 100 samples of 21 dogs with demodicosis, 1/20 (5%) cases were localized infections. In contrast, 20/80 dogs were in generalized form, resulting in an overall prevalence rate of 25%. The clinical signs of demodicosis-positive cases in street dogs were shown in Figure 3.

Alopecia, whole or partial hair loss, was seen in all 21 demodicosis-affected dogs (100%, 21/21), followed by white scale (66.70%, 14/21), erythema (61.90%, 13/21), pigmentation (52.38%, 11/21), pus-filled lumps (47.61%, 10/21), and hotspots (38.09%, 8/21) (Table 2). Distribution of clinical features and lesions on the forelegs and rear parts (tail and tail set) were the most common among the affected dogs, accounting for 42.85% (9/21) of instances each (Table 2).

The lesions were noticed in almost all parts of the bodies such that 66.6% (14/212) of the dogs had both shoulders and chest lesions, 28.6% (6/21) had lesions in the rear parts, and 42.9% (9/21) of the dogs had lesions in both the hindlimb and forelimb.

Hematological analysis revealed that there was no statistical significance between the control and demodicosis-positive groups of hematological data (Hb, RBC, platelets, PCV, MCV, and MCH) (*p* > 0.05, Table 3). The demodicosis-positive group had substantially lower mean corpuscular hemoglobin concentration (MCHC) (32.23 ± 1.66 mg/dL) than the control group (34.57 ± 2.24 mg/dL) (*p* = 0.010), whereas leucogram analysis showed no significant difference in WBC and monocyte counts between the demodicosis-positive group and control groups (*p* = 0.370 and 0.342). Leucogram analysis shows neutrophils were significantly higher in the demodicosis-positive group than in the control (*p* = 0.044), whereas lymphocytes were significantly lower (*p* = 0.033). Eosinophils were likewise significantly higher in the disease compared with the control (*p* = 0.004) (Table 3).

## 4. Discussion

The overall prevalence of canine demodicosis was recorded as 21% (21/100) in the present study. A similar prevalence of demodicosis was reported in other studies from neighboring areas in South Asian countries [14,23,24], which reported 27.78% (30/108) in India, 27% (27/100) in Bangladesh, and 29.1% (32/110) in Kathmandu, Nepal. However, this study’s findings were lower than the previous studies, which reported 4.9% (5/103) in the Republic of Korea and 10.5% (311/3055) in India [25,26]. In 2014, a survey conducted in Russia revealed demodicosis in 6.25% out of 157 domesticated dogs [27]. A 12-year-long study conducted at the Veterinary Parasitology Diagnostic Laboratory of Oklahoma State University examined the prevalence and trends of parasitic infections in client-owned dogs and found a low prevalence of canine *Demodex* infection at 0.22% (16/7409 cases), indicating that owned dogs may have a lower prevalence of *Demodex* mites [28]. Variations in the prevalence reported among research publications can be due to differences in source population, sample size variations, research duration, and management practices in different regions.

This study revealed *D. canis* associated with demodicosis in the study area resulting in 100% (21/21) prevalence. *D. canis* was associated with demodicosis which was slightly high in Taiwan where a 70.87% (73/103) prevalence of *D. canis* infection [29]. A case study of two canine demodicosis cases revealed the presence of *D. canis* [30]. Similarly, in India, morphometry analysis of mites collected from 32 positive cases revealed *D. canis* in all cases [31]. *D. canis* mites may live on the skin of healthy dogs, and the likelihood of identifying these mites is low [32]. *D. canis* may be more pathogenic or better suited to survival on canine hosts, resulting in higher infestation rates than other *Demodex* species.

The present study showed that prevalence varies by age category, with young dogs (<1 year) having the highest prevalence (37.5%, 3/8), followed by seniors (>6 years) (20%, 2/10) and adults (1–6 years) (19.5%, 16/82). Other studies also stated that dogs up to one year of age are more susceptible to demodectic mange (62.9%, 17/27) in Brazil [33] and (47.5%, 60%) in India [34], which was in close approximation to the findings of the present study. A study conducted in Bangladesh revealed a higher prevalence in young dogs (35%) than in adults (24.32%) and old dogs (17.39%) [24], and a study conducted in the UK found that 508 cases (72.4%) were diagnosed before 1 year of age [35]. Dogs aged 7–12 months had the highest occurrence (25.5%) of diseases [36]. Mites are attracted to sebum and are more likely to infest young dogs due to increased stimulation of their sebaceous glands throughout puberty [17]. At a particularly young age, canines are more susceptible to demodicosis referred to as “immunologic holes”. During this time, the animal’s natural host defenses, such as regular exfoliation and planned immunization, are weakened. Stressors such as changes in habitat, as well as physiological changes in the animal’s body, can contribute to this sensitivity [4].

According to this study, the gender-wise prevalence showed a slightly higher prevalence in females 21.56% (11/51 dogs) compared with males 20.40% (10/49 dogs). In India, it was reported that female dogs had a higher prevalence of demodectic mange (31.42%) than male dogs (26.02%) [23]. Other studies reported demodicosis being more prevalent in male dogs than in females [37,38]. Both male and female stray dogs may display similar behaviors and environmental exposures, resulting in a comparable prevalence of *Demodex* mites as the prevalence difference is barely 1.16% and statistically insignificant in the current study. Yet, the findings should explore more of the clinical aspects of animals.

Overall breed-wise prevalence of demodicosis in the study population revealed mixed/non-descriptive breeds showed the highest prevalence (33.33%, 3/9), followed by local ones (19.78%, 18/91), although it was not statistically significant. A study conducted in Kathmandu Valley revealed the highest prevalence of Mongrel (37.5%, 25/40), followed by pure (27.7%, 10/36), and mixed breed showed the lowest prevalence (25.9%, 7/34) [14]. Some studies revealed the highest prevalence in non-descriptive dogs (47.36%, 36.36%) [17,39], which contradicted the conclusion of another study that discovered that pure breeds were more susceptible to demodicosis than crossbreeds [40]. Mixed-breed dogs may have a more diverse genetic background, affecting their immune system’s capacity to fight against *Demodex* mites. As the sample size for mixed-breed dogs is considerably smaller, it can also result in more variability and less reliable prevalence estimations.

Dogs with over-ideal bodily conditions had a greater prevalence of demodicosis (25%, 4/16), followed by under-ideal (21.95%, 9/41) and ideal (18.6%, 8/43). Other studies reported similar findings, indicating that dogs in poor health were more susceptible to demodicosis than dogs in normal health [14,41]. However, this study’s results indicate that there is a little higher prevalence of demodicosis infection among dogs defined as above ideal in terms of bodily health than those classified as under ideal. Maybe obesity leads to skin folds that generate a warm and wet environment, which may be ideal for the proliferation of *Demodex* mites.

The prevalence of demodicosis according to its form/type revealed a higher prevalence in generalized form (25%, 20/80) than in localized form (5%,1/20). This was consistent with the study conducted in India where observed a higher prevalence of generalized demodicosis (36.06%, 45.46%) than localized demodicosis (17.02%, 23.46%), and both investigations found no significant association between clinical form and disease [23]. Experimental studies carried out in Ukraine revealed 24.6% demodectic cases with 30.1% of cases accounted for generalized and 69.9% for localized form [36]. The localized form typically affects up to four areas, often the face, neck, and forelegs, while the generalized form affects multiple or widespread lesions [42]. Generalized demodicosis may occur due to underlying health conditions or due to other factors that suppress the immune response toward mites’ proliferation. Delays in the diagnosis and treatment for a longer period of localized form may spread throughout the body, which ultimately progresses to a generalized form.

Alopecia was found in all the dogs investigated in this study, demonstrating a universal symptom. White scale, erythema, and pigmentation disorders were also common, affecting 66.66%, 61.90%, and 52.38% of the dogs, respectively. This finding is supported by a study conducted in India where it was reported alopecia in 100% of cases, followed by a white scale at 60% and erythema at 80% in a clinical evaluation of 21 demodicosis cases [43]. Alopecia was a persistent and notable clinical symptom displayed by almost all affected dogs, which may be caused by mite-induced inflammations and irritations in the hair follicles. The indications of the condition are related to irritation and inflammatory reactions created by mites in the hair follicles, resulting in damage to the epidermal cells and exudation produced by secondary bacterial and fungal infection [44]. The high prevalence of alopecia implies that it is a key clinical feature of canine demodicosis, although the existence of other symptoms such as erythema, pigmentation alterations, and pustules demonstrates the disease’s complexity.

Demodicosis-related lesions were distributed across various body parts, the foreleg and hind leg were the most affected, making up 42.9% of cases, while the shoulder and chest followed closely at 33.33%. The head and neck have significantly smaller percentages of affected individuals (19.04% and 23.80%, respectively). Lesions are widely dispersed, implying that *Demodex* infestation can impact numerous body regions rather than just one [43].

In this study, the hematological profile of affected dogs showed a significant reduction in MCHC associated with hemoglobin and a study conducted in Egypt showed a decrease in hemoglobin [45]. Skin lesion-induced anemia resulted in alterations in MCHC levels. This anemia could be due to the stress generated by the disease itself [2,46]. However, the leukogram showed significant elevation in neutrophils and eosinophils with a *p*-value of 0.044 and 0.004, while lymphocytes significantly decreased (*p*-value = 0.033). A study conducted in India found a similar increase in neutrophil and eosinophil counts, as well as a drop in lymphocyte counts, although it contrasted with an increase in WBC [17]. A study conducted in Egypt found a similar substantial increase and decrease in neutrophils and lymphocytes with *p*-values of 0.01 and 0.005 [47], while in India, it was discovered a decline in lymphocyte count with elevated eosinophils [18]. Lymphopenia was reported, which could be because cell-mediated immunity plays a crucial role in battling *Demodex* mites. Leukocytosis caused by neutrophils and eosinophils in infected animals may be related to prolonged antigenic stimulation and hypersensitivity reaction to *Demodex* persistence in tissues [17,48].

The present study’s limitations were primarily focused on micrometry for species identification. While micrometry is a valuable tool for morphological assessment in resource-poor settings, it may not capture the full genetic diversity or the subtle variations within species that molecular techniques like PCR could provide. Unfortunately, due to the current circumstances, PCR was not feasible, which restricts our ability to confirm species identity at a genetic level. This limitation may result in potential misidentifications or an incomplete understanding of the species’ ecological roles. Future studies could benefit from integrating molecular techniques alongside micrometry to enhance the accuracy and robustness of species identification. This study is limited by its sample size and geographical focus on Rupandehi, which may not fully represent demodicosis prevalence across Nepal. Further studies with a larger sample size, broader geographic coverage, environmental factors, and genetic susceptibility have to be carried out to assess the impact of demodicosis in Nepalese street dogs.

## 5. Conclusions

The current research revealed the incidence of canine demodicosis caused by *D. canis* with 21% positivity among street dogs of Rupandehi, Nepal. The most common rates of infestation were recorded in young puppies (37.5%), females (21.6%), crossbreeds (33.3%), and dogs having more than ideal body condition (25%). Alopecia was a prominent clinical sign, predominantly on the legs. Hematological analysis of demodicosis-positive dogs showed significant increases in neutrophils and eosinophils and a significant reduction in MCH concentration and lymphocytes. The statistical analysis revealed no significant association (*p* > 0.05) between demodicosis positivity and factors such as age, gender, breed, or nutritional status. However, the clinical significance of the suffering due to demodicosis in street dogs should not be undermined because the findings of this study could be guidance for local veterinarians in the management of canine demodicosis. This study highlights the importance of diligent monitoring and comprehensive diagnostic tests in managing and treating canine demodicosis effectively and emphasizes the need for better veterinary care and preventive measures to control demodicosis, particularly in the vulnerable street dog populations of Nepal.

## Figures and Tables

**Table 1:** Prevalence of canine demodicosis in street dogs based on different parameters.

Animal Characteristics	Sub Characters	Total Samples Collected	Positive	Negative	Prevalence [Positive Cases (%) (95% CI)]	OR (95% CI)	Fisher Exact *p*-Value
Breed	Local	91	18	73	19.78 (12.89–29.11)	Ref	Ref
	Mixed breed	9	3	6	33.33 (12.89–29.11)	1.65 (0.53, 8.64)	*p* = 0.39
Age (years)	Young (<1)	8	3	5	37.50 (13.68–69.42)	Ref	Ref
	Adult (1–6)	82	16	66	19.50 (12.38–29.37)	1.94 (0.60, 10.87)	*p* = 0.36
	Senior (>6)	10	2	8	20 (5.66–50.98)	1.33 (0.31, 15.14)	*p* = 0.61
Sex	Male	49	10	39	20.40 (11.48–33.64)	Ref	Ref
	Female	51	11	40	21.56 (12.49–34.63)	0.95 (0.42, 2.75)	*p* = 1.0
Nutritional status	Under Ideal	41	9	32	21.95 (12.00–36.71)	Ref	Ref
	Ideal	43	8	35	18.60 (9.74–32.62)	1.23 (0.41, 3.69)	*p* = 0.79
	Over Ideal	16	4	12	25 (10.20–49.49)	0.83 (0.22, 3.69)	*p* = 1.00

OR, odd ratio; CI, confidence interval; and Ref, references.

**Table 2:** Clinical findings of canine demodicosis (*n* = 21) in different body parts of street dogs.

Variables	Category	Number of Demodicosis Cases *n* (%)
Clinical features and lesions	Alopecia	21 (100.0)
	White scale	14 (66.7)
	Erythema	13 (61.9)
	Pigmentation	11 (53.4)
	Pustular	10 (47.6)
	Hotspot	8 (38.0)
Site of lesion noticed	Forelimb	9 (42.9)
	Hind limb	9 (42.9)
	Shoulder and chest	14 (66.6)
	Rear parts	6 (28.6)
	Whole body *	6 (28.6)
	Neck and head	9 (42.9)

Skin lesions on the whole body of the dogs. * lesion noticed all body parts

**Table 3:** Hematological analysis of healthy and demodicosis-affected dogs (Mean ± SD).

Parameters	Control or Healthy	Diseased	Reference Value	*p*-Value
Hemoglobin (g/dL)	13.57 ± 1.66	12.74 ± 1.95	12–19	0.23
RBC (million/μL)	6.01 ± 0.52	6.22 ± 1.44	5.6–8.1	0.55
Platelets (1000/μL)	234 ± 64.30	220.04 ± 132.09	211–621	0.69
PCV (%)	39.33 ± 5.00	40.007 ± 7.021	37–57	0.76
MCV (fl)	65.39 ± 5.76	64.45 ± 8.79	60–77	0.72
MCH (pg)	22.61 ± 2.22	21.13 ± 3.33	19–24	0.15
MCHC (mg/dL)	34.57 ± 2.24	32.23 ± 1.66	31–36	0.01 *
WBC (counts/μL)	12,230 ± 3756.78	13,770 ± 5492.03	5000–16,000	0.37
Neutrophils (%)	64 ± 10.40	72.22 ± 8.09	58–85	0.04 *
Lymphocytes (%)	32 ± 10.31	23.36 ± 7.74	8–21	0.03 *
Monocytes (%)	2.5 ± 1.07	3.033 ± 1.98	2–10	0.34
Eosinophils (%)	1.5 ± 1.17	3.38 ± 2.19	0–9	0.01 *

RBC, red blood cells; PCV, packed cell volume; MCV, mean corpuscular volume; MCH, mean corpuscular hemoglobin; MCHC, mean corpuscular hemoglobin concentration; WBC, white blood cell. * *p*-Value statistically significant.

## Data Availability

The datasets generated and/or analyzed during the current study are available in the manuscript.

## Supplementary Materials

The following supporting information can be downloaded at https://www.mdpi.com/article/10.3390/vetsci12030238/s1, Table S1: Morphometric analysis of *D. canis*.
