# Supplementary material for: Canine Demodicosis in Rupandehi Nepal’s Street Dogs: Prevalence, Clinical Signs, and Hematology
Source: Vet Sci. 2025 Mar 3;12(3):238. doi: 10.3390/vetsci12030238 (PMC11946785; doi:10.3390/vetsci12030238)
Supplement: Supplementary file 1 [file vetsci-12-00238-s001.zip › vetsci-3476840-supplementary.pdf]

Supplementary Table S1: Morphometry analysis of *D. canis*

**Parameters**		**Micrometry of *D. canis* (n=21)**		**Shivajothi et al, 2024**
		Average	Range	*D. canis*
Gnathosoma	L (μm)	18.11±1.4	15.2-20.1	20.87±0.87
	W (μm)	16.95±1.6	13.5-19.2	19.91±0.31
Podosoma	L (μm)	63.03±1.07	60.7-64.7	63.18±0.67
	W (μm)	38.35±2.03	32.4-42.4	38.92±0.28
Opisthosoma	L (μm)	130.89±4.18	119.8-140.1	132.71±2.66
	W (μm)	34.11±3.98	25.9-39.6	34.82±0.18
Total body length	L (μm)	212.04±5.12	197-219.8	216.67±4.33
The ratio of the length of opisthosoma to body length	(%)	61	60-63	

L, length; W, width
